# Consumer Engagement With Risk Information on Prescription Drug Social Media Pages: Findings From In-Depth Interviews

**DOI:** 10.2196/67361

**Published:** 2025-03-25

**Authors:** Jacqueline B Amoozegar, Peyton Williams, Kristen C Giombi, Courtney Richardson, Ella Shenkar, Rebecca L Watkins, Amie C O'Donoghue, Helen W Sullivan

**Affiliations:** 1 RTI International Research Triangle Park, NC United States; 2 US Food and Drug Administration Silver Spring, MD United States

**Keywords:** social media, prescription drugs, risk information, safety information, Facebook, Instagram, prescription, risk, information, safety, interview, consumer engagement, digital, drug promotion, user experience, promotion

## Abstract

**Background:**

The volume of digital drug promotion has grown over time, and social media has become a source of information about prescription drugs for many consumers. Pharmaceutical companies currently present risk information about prescription drugs they promote in a variety of ways within and across social media platforms. There is scarce research on consumers’ interactions with prescription drug promotion on social media, particularly on which features may facilitate or inhibit consumers’ ability to find, review, and comprehend drug information. This is concerning because it is critical for consumers to know and weigh drug benefits and risks to be able to make informed decisions regarding medical treatment.

**Objective:**

We aimed to develop an understanding of the user interface (UI) and user experience (UX) of social media pages and posts created by pharmaceutical companies to promote drugs and how UI or UX design features impact consumers’ interactions with drug information.

**Methods:**

We conducted in-person interviews with 54 consumers segmented into groups by device type (laptop or mobile phone), social media platform (Facebook or Instagram), and age. Interviewers asked participants to navigate to and review a series of 4 pages and 3 posts on their assigned device and platform. Interviewers encouraged participants to “think aloud,” as they interacted with the stimuli during a brief observation period. Following each observation period, participants were asked probing questions. An analyst reviewed video recordings of the observation periods to abstract quantitative interaction data on whether a participant clicked on or viewed risk information at each location it appeared on each page. Participants’ responses were organized in a metamatrix, which we used to conduct thematic analysis.

**Results:**

Observational data revealed that 59% of participants using Facebook and 70% of participants using Instagram viewed risk information in at least 1 possible location on average across all pages tested during the observation period. There was not a single location across the Facebook pages that participants commonly clicked on to view risk information. However, a video with scrolling risk information attracted more views than other features. On Instagram, at least half of the participants consistently clicked on the highlighted story with risk information across the pages. Although thematic analysis showed that most participants were able to identify the official pages and risk information for each drug, auto-scrolling text and text size posed barriers to identification and comprehensive review for some participants. Participants generally found it more difficult to identify the drugs’ indications than risks. Participants using Instagram more frequently reported challenges identifying risks and indications compared to those using Facebook.

**Conclusions:**

UI or UX design features can facilitate or pose barriers to users’ identification, review, and comprehension of the risk information provided on prescription drugs’ social media pages and posts.

## Introduction

### Background

Pharmaceutical companies are present and active on social media. Their multipurpose use of social media platforms for corporate identity, community management, and paid promotion of their products achieves varying levels of measured success [1]. The health care and pharmaceutical industry in the United States spends a significant amount of money on social media promotion. Between July and November 2020, this industry spent US $198.3 million on Facebook promotion and US $151.5 million on Instagram posts [2]. The size of these expenditures is particularly noteworthy, given that the cost of digital promotion is considerably lower than other mediums (eg, television).

Consumers can use social media to find information about prescription drugs in a variety of ways, including searching for information from pharmaceutical companies, reading reviews and testimonials, and interacting with other consumers. A nationally representative survey of 1744 US adults found that 26% of respondents had seen a prescription drug advertised on social media in the past 3 months, and 9% had watched a video on the web about a prescription drug on a social networking website [3]. Another survey of 1000 US consumers found that 9% used social media to evaluate new treatment options, and 7% used social media to look for reliable information about medication side effects [4]. A systematic review found that consumers use social media as a complement to a physician’s treatment, particularly around social, emotional, esteem, network, and information support [5].

Despite these increases in the volume of digital drug promotion, the amount of research on consumers’ use of and trust in prescription drug promotion on social media is scarce, particularly on user interface (UI) and user experience (UX) features associated with accurate understanding of product claims and risks. This lack of evidence is problematic, given that the information shared on these platforms could have a large impact on the treatment decisions of consumers. For example, when this information is shared on television, one study found that exposure to prescription drug promotion for as little as 5 minutes increased consumers’ intentions to search for medications on the web or switch medications [6]. Another study found that reading a prescription drug promotion on Twitter was associated with college students obtaining a prescription medication without first obtaining a prescription from a doctor [7].

Prescription drug promotion is regulated by the Food and Drug Administration [8]. According to Food and Drug Administration regulations, when drug benefits are promoted, drug risks must also be described to achieve a “fair balance” [9]. Ensuring “fair balance” on an interactive medium like social media—where there could be character space limitations, targeted newsfeed advertisements, and consumer interaction through comments, shares, and likes—is challenging.

Pharmaceutical companies currently present risk information (often referred to as “safety information” in promotion) about the prescription drugs they promote in a variety of ways across social media platforms. For example, companies may use links to provide access to risk information, such as a link to the drug’s external website or a link to a PDF file with the full prescribing and medication guide. Risk information is also sometimes conveyed in a featured text post, a scrolling video post, a risk information pop-up, or a highlighted story.

Many pharmaceutical companies place the risk information in multiple places on their social media pages and posts. However, even when this information is displayed in various locations, UI or UX features of social media platforms may still inhibit consumers’ ability to find, review, and comprehend the risk information. This is concerning, given that it is critical for consumers to know and weigh drug benefits and risks to be able to make informed decisions regarding their medical treatment.

### Study Purpose and Value

With the increasing use of social media for promotions, understanding the UI or UX design and its impact on how consumers interact with the pages and posts created by pharmaceutical companies to promote drugs is becoming increasingly important. Accordingly, we examined how different approaches to presenting information about the risks of promoted drugs on social media pages and posts influence how consumers engage with the content. Specifically, we assessed how consumers’ exposure to and perceptions of risk information was influenced by where and how on the social media pages and posts the information was conveyed. Further, we sought to identify differences in experiences and perceptions by user age as well as the type of device being used to access the pages and posts.

## Methods

### Study Design

We developed our plan and instruments for conducting in-person, one-on-one interviews with a total of 54 individuals based on our research questions and a review of relevant peer-reviewed literature. As shown in Table 1, study participants were segmented based on three criteria: (1) device type, (2) social media platform, and (3) age. For device type, we segmented participants into reviewing stimuli on either a mobile device (an Apple iPhone 6s running iOS 15.8) or a laptop computer (an Acer Aspire E5-571 laptop with a 15.6-inch screen running Windows 10). For social media platform, we segmented participants to review stimuli on either Facebook or Instagram. For age, participants in the Facebook segment were further segmented into either “adults” (18-61 years of age) or “older adults” (62 years of age or older). Participants in the Instagram segment were further segmented into either “younger adults” (18-24 years of age) or “adults” (25 years of age or older). The different definitions of “adults” for Instagram versus Facebook reflect that users of Instagram are typically younger [10].

**Table 1:** Sample segmentation by device type used, social media platform used, and age.

	Device type			Social media platform			Age cohort		
	Mobile	Laptop	Facebook		Instagram	18+ years		18-24 years	62+ years
Group 1 (n=9)	✓		✓			✓			
Group 2 (n=9)		✓	✓			✓			
Group 3 (n=9)	✓				✓	✓			
Group 4 (n=9)	✓				✓			✓	
Group 5 (n=9)	✓		✓						✓
Group 6 (n=9)		✓	✓						✓

### Recruitment and Screening Process

To recruit participants, we partnered with a marketing research firm with access to a consumer panel with a national population. This firm emailed adult panelists an invitation to participate. Interested panelists clicked on a hyperlink within the invitation to complete a web-based screener to determine eligibility. If deemed eligible by the web-based screener, participants were contacted by phone to complete a more comprehensive screening process.

To be eligible, participants needed to use either Facebook or Instagram 4 or 5 days per week on a smartphone or laptop or desktop. Participants also had to self-report a type 2 diabetes diagnosis by a medical professional to increase the likelihood that participants would be motivated to learn about some of the products included in the stimuli, which would in turn increase the realism and thus how generalizable our study findings are to the real world and everyday life. We encountered difficulties recruiting persons with type 2 diabetes for the 18- to 24-year age group assigned to view Instagram stimuli on mobile devices (group 4) and removed the diabetes requirement for this one group as a result.

Because the interviews were conducted in person, participants needed to be able to drive or otherwise be transported to the study location (a private meeting room in an office setting) in Raleigh, North Carolina. To reduce the risk of bias, we excluded participants who worked in marketing, in the pharmaceutical industry, or for the US Department of Health and Human Services. Individuals who had participated in a focus group or interview-based research study in the previous 3 months were also ineligible to participate.

### Ethical Considerations

This study was determined to be exempt under category 2ii (tests, surveys, interviews, or observation) by RTI International’s institutional review board (STUDY00022581) on August 14, 2023. Eligible participants were sent a consent form in advance of the interview providing information about the protection of their privacy and the confidentiality of their data. Additionally, the interviewer reviewed the information presented in the consent form before starting each interview and obtained the participant’s verbal consent to participate and have their voice and screen interactions recorded. Participants were informed that neither the transcripts produced based on interview recordings nor any reporting of findings would include identifying information. No potential participants declined to consent or dropped out after consenting. Upon completion of each interview, the participant was sent a US $75 incentive in the form of a digital gift card.

### Stimuli

We identified existing social media pages and posts created by pharmaceutical companies for the prescription drugs they manufacture. We focused on Facebook and Instagram pages and posts based on use trends and to limit the number of combinations of variables to be assessed [10].

We selected pages and posts about prescription drugs with a mix of indications, including some that were relevant, and possibly familiar, to many of our participants (ie, drugs indicated to treat diabetes) and some that were not (eg, drugs indicated to treat high cholesterol). To avoid endorsing specific products, we have anonymized the stimuli and referred to the pages and posts by numbers (ie, rather than the promoted drugs’ brand names).

We began each interview by asking all participants to review the same “baseline” stimuli page for one of the selected prescription drugs not indicated to treat diabetes (with which they were unlikely to be familiar) with the hope that they would have to explore the content in more depth to learn about the indications, risks, and benefits of a drug that are novel to them.

In total, 3 pages and 3 posts for each of the 2 platforms (ie, Facebook and Instagram) served as the remainder of our stimuli. Key characteristics of these pages and posts are shown in Tables 2 and 3, respectively. Working within the confines of selecting only real-life pages and posts, we sought to achieve the best possible variation in stimuli. For instance, for Facebook pages, we sought pages where engagement policies varied (ie, whether consumers were allowed to comment on or react to posts). For posts, we strove for variation in how risk information was shown, such as whether it appeared in static text or scrolling text in a video.

**Table 2:** Key characteristics of the study stimuli: prescription drug–focused pages on Facebook and Instagram.

Characteristic	Facebook					Instagram			
	Baseline page	Page #1	Page #2	Page #3	Baseline page		Page #1	Page #2	Page #3^a^
Condition	Multiple autoimmune disorders	Diabetes	Diabetes	High cholesterol	Multiple autoimmune disorders		Diabetes	Diabetes	High cholesterol
Audience engagement allowed on posts	No	Yes	No	No	No		Yes	Yes	Unknown (content removed)
Verified account	Yes	Yes	Yes	Yes	Yes		No	No	Yes

^a^We excluded this page from our analysis because all content (except the risk information pinned post and an external link) was deleted from the page between when we selected it and when we started testing. With limited places to click, all participants who viewed this page clicked on the risk information highlighted story.

**Table 3:** Key characteristics of the study stimuli: prescription drug–focused posts on Facebook and Instagram.

Characteristic	Facebook			Instagram		
	Post #1	Post #2	Post #3	Post #1	Post #2	Post #3
Type of post	Video	Text only	Video	Video	Photo	Video
Condition	Diabetes	Diabetes	High cholesterol	Diabetes	Diabetes	Diabetes
Risk information is included	Yes	Yes	Yes	Yes	Yes	Yes
Post focuses exclusively on risk information	Yes	Yes	No	No	No	No
Risk information is scrolling	Yes (entire frame scrolls)	No	Yes (bottom one-third of video scrolls)	No	N/A^a^	Yes (some information appears in static text with voice-over; additional information scrolls with no voiceover)
Risk information is shown immediately in the video	Yes	N/A	Yes	No	N/A	Yes (a note advises viewer that safety info appears at the end of the video)
Risk information is shown in caption	No	N/A	Only a hyperlink is shown in caption	No	Yes	Yes
Must click “See more” to view all warnings	N/A	N/A	N/A	No	Yes	Yes
Allows comments on posts	No	Yes	No	Yes	Yes	Yes

^a^N/A: not applicable.

### Data Collection

We conducted 54 in-depth, in-person interviews using a semistructured interview guide during October and November 2023. Each interview lasted approximately an hour and was conducted by 1 of 3 experienced qualitative researchers (PW, KCG, and RLW) employed by an independent, nonprofit research institute.

Based on the participant’s assigned group, the interviewer asked each participant to use the study mobile phone or laptop to navigate to stimuli on the assigned social media platform. A camera was positioned above the phone screen to record the surface of the phone as well as the fingers of the participants and where they were tapping on the phone. For both devices, the screen was shared, recorded, and livestreamed via Zoom (Zoom Video Communications) to enable the other members of the research team to observe.

First, interviewers directed participants to the baseline drug’s page on Facebook or Instagram (depending on the participant’s assigned segment) via the platform’s search function. Using a think-aloud approach, interviewers encouraged participants to narrate their thoughts and feelings on the social media content as they interacted with the study stimuli [11]. The interviewers allowed participants to explore the page and posts on their own for 3 to 5 minutes, which we called the “observation period.” After the observation period, the interviewers asked probing questions.

Second, participants reviewed the 3 subsequent drugs’ pages. Again, the interviewers allowed an observation period for each page before asking probing questions. After participants had looked at all the pages, the interviewers guided participants to navigate to predetermined posts. The interviewers shuffled the order of the stimuli pages and posts to be reviewed between interviews (with the exception of the baseline stimuli page) so that the sequence was sufficiently random to prevent order effects.

### Quantitative Analysis

After data collection was complete, an analyst watched the video recordings of the screenshare to abstract interaction data collected during the observation period. We only abstracted interaction data from the observation period, given that this was the only time participants were able to freely explore the pages on their own.

Because risk information could appear in multiple locations, we identified all locations within each of the stimuli where risk information was displayed or linked before beginning data abstraction. Locations varied greatly across stimuli and included approaches such as links to PDFs, videos that displayed risk information, and posts with static written risk information.

During data abstraction, the analyst recorded whether a participant clicked on or viewed risk information at each location. We calculated the following metrics:

- Ever attentive: For a given drug’s social media page, risk information could be shown in multiple locations. “Ever attentive” is the percentage of participants who viewed the risk information for a given drug’s page in at least 1 of those locations during the observation period. For example, 10% ever attentive means that 10% of participants viewed risk information in at least 1 location for a given drug’s page. This metric helped us determine whether consumers see and engage with information about risks.
- Locations viewed: This metric is more granular and helps us understand the exact locations participants viewed the risk information. “Locations viewed” is the percentage of participants who viewed risk information at a particular location or feature. This could have involved actively clicking on a link or been more passive, such as having the risk information appear upon scrolling down. This metric helped us identify the features or characteristics of the platform associated with consumers’ review of risk information.

When abstracting data, we did not code the participant’s interactions with social media content for the final page that they reviewed. We opted for this approach because we noticed that most participants learned over the course of the interview that we would be asking them about the drugs’ risk information and began proactively seeking this information upon switching to a new stimulus in preparation to answer our questions. Similarly, we did not code participants’ interactions with the predetermined posts that participants navigated to after reviewing the final page.

### Qualitative Analysis

During each interview, we entered deidentified participant responses into a metamatrix that organized responses by participant ID, interview topic, and segment (ie, device type, platform, and user age). We selected a metamatrix approach because this usability study used interview notes (which were most efficiently captured in Microsoft Excel by notetakers) as our unit of analysis (ie, vs using the transcripts themselves). Unlike the transcripts, the notes captured observational data reflecting what notetakers saw when the user interacted with the pages and posts (eg, the user scrolled past relevant information or clicked around to locate information). The metamatrix enabled us to see multiple participant answers across a single topic and question, which facilitated the identification of themes or consistencies in responses [12].

Following data collection, 3 analysts conducted a thematic analysis to identify commonalities and patterns and to summarize the findings for all pages and posts [13]. Organizing the matrix by segment facilitated the identification of any differences between the segments. Given the sample size and the fact that participant responses were not formally coded using computer software, qualitative themes were not associated with participant counts. A general sense of magnitude is included by describing what portion of participants mentioned each theme (eg, most participants, many participants, and a few participants) [14,15].

## Results

### Participant Characteristics

The 54 consumers who participated in interviews were reasonably varied with regard to demographic characteristics. Most study participants self-identified as male (n=33, 61%). Of the 36 participants assigned to view Facebook stimuli, 44% (n=16) were in the “adult” segment (18-61 years of age), and 56% (n=20) were considered “older adults” (62 years of age or older). The 18 participants assigned to view Instagram stimuli were evenly split into the “younger adult” (18-24 years of age) and “adult” (25 years of age or older) segments. The mean age for participants viewing Facebook stimuli (60.1, SD 12.16 years) was approximately 22 years older than the mean age for participants viewing Instagram stimuli (38.1, SD 18.05 years). Most of the participants were White (n=39, 72%), and nearly one-fourth (n=13, 24%) were Black or African American. Finally, most participants held an undergraduate or postgraduate degree (n=32, 60%).

### Key Quantitative Findings

Results of the analysis of our quantitative, observational data are presented for each of the 2 metrics described in our quantitative analysis methods earlier (ie, ever attentive and locations viewed) and further organized by platform. Differences by device type have been noted as appropriate.

#### Ever Attentive to Risk Information

##### Facebook

On average, across all the drugs’ pages that we tested on Facebook, 59% of participants were ever attentive to risk information during the observation period (Table 4). Of interest, a higher proportion of the participants using the laptop (72%) were attentive to risk information compared to less than half of the participants using the mobile phone (46%).

**Table 4:** Participants who were ever attentive to risk information by platform, device, and prescription drug page.

Platform and device			Baseline page, n (%)		Page #1, n (%)		Page #2, n (%)		Page #3, n (%)		Average (%) (SD)
**Facebook**											
	Overall	18 (50)		15 (71)		19 (63)		11 (55)		59 (3.99)	
	Laptop	10 (56)		8 (80)		12 (86)		8 (73)		72 (5.62)	
	Mobile	8 (44)		7 (64)		7 (44)		3 (33)		46 (5.59)	
**Instagram**											
	Mobile	18 (67)		14 (79)		15 (67)		N/A^a^		70 (3.27)	

^a^N/A: not applicable.

##### Instagram

On average, across all the drugs’ pages that we tested on Instagram, 70% of participants were ever attentive to risk information. Notably, a higher proportion of participants (70%) viewing Instagram pages on a mobile phone were ever attentive to information about risks as compared to Facebook mobile (46%).

#### Risk Information of Locations Viewed

##### Facebook

**Figure 1:**
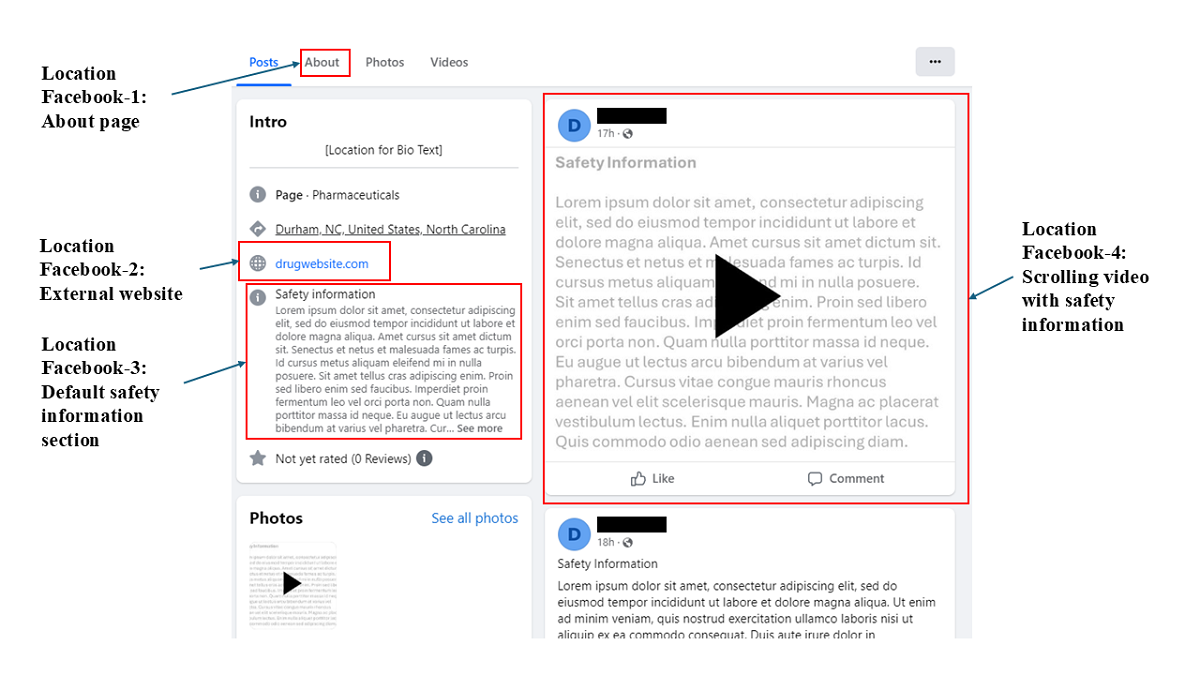
Common locations of risk information on Facebook pages about prescription drugs (as it appears on a laptop).

Each Facebook page we tested had nuances in how and where the pharmaceutical company presented risk information, making it difficult to summarize commonalities and differences among them. We did, however, observe 4 common locations where pharmaceutical companies displayed risk information on Facebook (Figure 1).

- Location Facebook-1: “About” page. This page was accessed by clicking on “About” from the “Posts” page. If a page is set up as a “Pharmaceuticals” page, Facebook automatically gives the page creator the ability to activate a “Safety Information” section on the “About” page. Risk information was frequently placed here, although it is not required by Facebook.
- Location Facebook-2: external website. On the “Posts” page, pharmaceutical companies often included a link to their drug’s external website. Companies frequently display risk information at the bottom of their external website landing page.
- Location Facebook-3: default “Safety Information” section in the introduction pane. By default, once a pharmaceutical company adds the “Safety Information” section to the “About” page, Facebook then adds a section entitled “Safety Information” to the introduction pane on the “Posts” page. All text that is added in location Facebook-1 is automatically duplicated here. If a participant clicks “See More,” a pop-up will appear with the full text.
- Location Facebook-4: scrolling video. Pharmaceutical companies frequently create videos of scrolling textual risk information.

We expected location Facebook-3—the “Safety Information” section in the introduction pane—to have a consistently high level of engagement because it is clearly labeled, but we were surprised to find a wide range in the proportion of participants who viewed it across the drugs (Table 5).

A video with scrolling risk information seemed to be the location most often viewed with risk information. For Facebook on a laptop, at least 40% (n=4) of participants consistently viewed risk information when it was presented in a scrolling video, potentially because the motion caught their attention. On the other hand, the percentage of participants who viewed the scrolling video on the mobile device was lower across the 3 pages using this approach (between n=2, 11% and n=4, 36%).

We found that if 2 or more links to external websites were presented together, participants often only clicked the first link. For example, in situations where links to the full prescribing information and risk information appeared one after another, participants who engaged with either link would always click the first one, but not the second. Furthermore, when the link went to the full prescribing information, participants did not find the risk information or medication guide in the PDF.

**Table 5:** Participants who viewed risk information on Facebook and Instagram by common location on prescription drug–focused pages.

Location and device				Baseline page, n (%)		Page #1, n (%)		Page #2, n (%)		Page #3, n (%)
**Facebook**										
	**Location Facebook-1: “About” page**									
		Laptop	N/A^a^		2 (20)		3 (25)		0 (0)	
		Mobile	N/A		2 (18)		1 (6)		1 (11)	
	**Location Facebook-2: external website**									
		Laptop	2 (11)		0 (0)		3 (25)		5 (45)	
		Mobile	6 (33)		3 (27)		5 (31)		3 (33)	
	**Location Facebook-3: default “Safety Information” section in the introduction pane of the “Posts” page**									
		Laptop	N/A		2 (20)		8 (67)		1 (9)	
		Mobile	N/A		1 (9)		8 (50)		0 (0)	
	**Location Facebook-4: scrolling video**									
		Laptop	10 (56)		4 (40)		N/A		6 (55)	
		Mobile	2 (11)		4 (36)		N/A		3 (33)	
**Instagram**										
	**Location Instagram-1: external website link**									
		Mobile	9 (50)		4 (27)		8 (57)		N/A	
	**Location Instagram-2: highlighted story with risk information**									
		Mobile	9 (50)		9 (60)		7 (50)		N/A	

^a^N/A: not applicable.

##### Instagram

**Figure 2:**
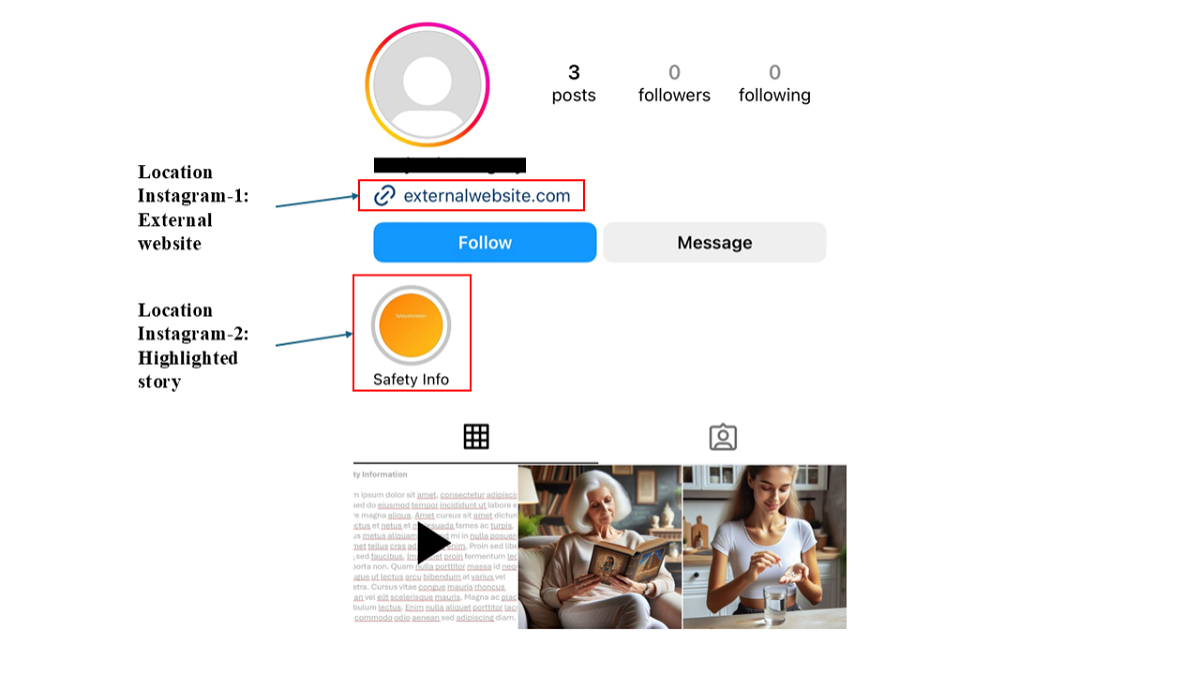
Common locations of risk information on Instagram pages about prescription drugs.

Unlike Facebook, Instagram does not have a reserved section dedicated to risk information. This means that pharmaceutical companies must find alternative ways of displaying risk information. There were 2 main locations that pharmaceutical companies used to convey this information on the profile page, which is the main page a person reaches when visiting a drug’s Instagram page (Figure 2).

- Location Instagram-1: external website. Instagram allows one or more external links to be included on the profile page.
- Location Instagram-2: highlighted story with risk information. Every drug page on Instagram that we tested created a highlighted story with risk information. A highlighted story is a story that is pinned at the top of the page. These stories can have one or more screens.

Location Instagram-2—highlighted stories—was a common place for participants to view risk information. For all 3 drugs’ pages, at least half of the participants consistently clicked on the highlighted story with risk information.

### Key Qualitative Findings

Results from our analysis of participants’ qualitative interview responses are presented by topic: (1) identification of official pages and verified accounts, (2) ease of locating risk information, (3) factors affecting comprehensiveness of review of risk information, and (4) identification of product risks and indications. Quotations illustrating key themes have been included as appropriate.

#### Identification of Official Pages and Verified Accounts

Regardless of platform, the majority of participants were able to identify the official page for each drug when searching for it without assistance from the interviewer. Some participants reported that they could tell the page was official because they saw a registered trademark symbol. Participants also reported relying on various other indicators such as the presence of a blue checkmark, the number of followers, the number of posts, and the pharmaceutical company’s branding (ie, colors and logos). Participants further mentioned noticing details such as the generic name of the drug, external links to pharmaceutical company websites, and specific medical terms (eg, glucagon and insulin). As one participant said, “It has a Linktree, a lot of followers, and they have 420 posts, which makes me think it’s official” [Instagram, mobile, adult]. Finally, another sign participants viewing pages on Facebook noticed was the “Pharmaceuticals” page type in the profile.

When asked about the meaning of the blue checkmark next to the drug’s name, participants’ responses were mixed. Some participants were unsure about its meaning. Others explained that the blue checkmark meant that the page was real and legitimate or that it confirmed that the page’s creator is indeed who they say they are. One participant described their interpretation of the checkmark’s implications as: “It’s not somebody else with their opinion of it; it’s more sort of facts about the drug as the drug company sees it based on the negative reactions that the drug companies had, and they’ve done their studies” [Facebook, mobile, older adult]. Most participants who reported uncertainty about the meaning of the blue checkmark were older adults viewing Facebook pages.

Participants’ responses were also mixed about whether an account being verified influenced their level of trust in the information provided on the page. Some participants reported that account verification has no effect on how much they trust the information. Older adults who viewed Facebook pages were more likely to report that an account being verified influenced their trust of the information compared to adults in the younger age segment. Similarly, none of the young adults who viewed Instagram pages reported that the account being verified affected their trust of the information. One participant viewing Instagram stimuli explained their lack of trust in the blue checkmark: “It doesn’t seem like an actual verification since you can pay for it!” [Instagram, mobile, adult].

#### Ease of Locating Risk Information

As participants finished reviewing each page or post, we asked how difficult it was to locate the risk information for the drug. Participants commonly reported that it was easy to find the risk information because the information appeared in multiple places or near the top of the page or post. One young adult participant viewing Instagram stimuli explained that it was “not hard, just because it was pinned at the top, and typically things that are pinned are most important” [Instagram, mobile]. However, some participants did find the risk information challenging to locate or at least reported that it took more effort to find information on these drug pages than on other types of pages. We noticed frustration when participants clicked on a link where they expected to find patient safety information but instead found the full prescribing information (ie, information intended for a clinical audience). Furthermore, participants noted at times, especially with third-party multilink services, that they had difficulty finding risk and patient-oriented information.

We asked participants to identify ways this important information could be made easier to find. Participants offered multiple ideas, including changing the formatting of the information by using bold, highlighted, colorful, or larger text; applying section headers or other descriptive labels clearly identifying the topic; using pinned or featured posts; adding risk information as an option in the menu bar (Facebook); and including links in additional places.

#### Factors Affecting Comprehensiveness of Review of Risk Information

After viewing each of 3 consecutive posts on their assigned platform, participants were asked whether they would normally watch or read the entire post. Participants’ responses were mixed across platforms and posts. Participants who reported that they would normally watch or read the entire post commonly clarified that it would depend on whether they were interested in the specific drug highlighted in the post. Some participants who reported that they would not watch or read the entire post indicated that they would just skim it for specific information. Of those who said they would skip the post entirely, a few said they would learn about the drug from an information source other than social media (eg, the drug’s website and their physician).

We asked participants who viewed the posts on Facebook whether they were more or less likely to read all the information in the post if it was auto-scrolling and whether they would prefer the post to scroll automatically or not. A few participants reported that they preferred scrolling information (and claimed they would be more likely to read it) or that they did not have any preference about scrolling. However, participants more commonly reported being less likely to fully read scrolling risk information because they prefer to read at their own pace or because they found the speed of scrolling or font size of the scrolling information to be less than ideal. Although most participants knew how to pause a scrolling video, they preferred to avoid the need for pausing or rewinding, especially if they were seeking specific pieces of information. One participant who said that they were less likely to read scrolling information explained, “I don’t like the scrolling. I want to go at my speed. If I don’t have issues with my gallbladder, I want to skip that information. I don’t want to read about gallbladder issues” [Facebook, mobile, adult]. Another participant who reacted negatively to scrolling indicated, “When I saw the scrolling, I saw the pace which I thought was too fast and then I thought, is there something else that could help me? And I saw the links ...” [Facebook, computer, older adult].

After viewing all the Instagram posts and their variety of approaches to showing risk information, participants were asked which they were most and least likely to fully read. Of the posts, 2 presented risk information in static text; participants typically reported one of these 2 posts as being the one they were most likely to read fully. Beyond aversions to scrolling, participants also cited the volume and quality of information as well as how concisely it was written, as factors affecting the likelihood that they would fully review it.

#### Identification of Product Risks and Indications

After reviewing the baseline drug’s page, participants were asked if they felt that they knew the drug’s risks and indications. Regardless of whether they viewed the page on Facebook or Instagram, most participants were able to locate the drug’s risks without any difficulty. Participants commonly demonstrated that they had successfully found the risks by reading several of them aloud. Some participants clarified that they would need to fully review the risk information to feel like they really “knew” the risks. A few acknowledged that they had not reviewed the page very closely when perusing the drug information.

Similarly, when reviewing the subsequent drugs’ pages, nearly all participants indicated that they were able to find the risk information easily and felt that they knew the risks of the drugs. We also asked participants to imagine that they were worried about a specific side effect and to explain whether they would know where to look to find this information. Almost all participants demonstrated that they knew where to find information about the specified side effects.

We asked participants what the risks of a given drug were. Instagram users more frequently reported that they did not know the risks compared to Facebook users. Of the participants who reported that they did not feel that they knew, or could easily identify, the drugs’ risks, scrolling and small text size were commonly cited as reasons irrespective of platform. One participant viewing Facebook stimuli remarked, “If I could read this little fine print here, it’s probably got it all in there” [Computer, older adult]. Other explanations specific to Instagram included the perception that the drug pages were uninformative and issues with risk information being obscured by other page elements.

Participants were also asked if they felt they knew “what the drug is for” (ie, its indications). Overall, participants generally experienced more difficulty finding this information than they had in finding the risks. However, most participants were ultimately successful in identifying the indications or at least in identifying where they could obtain that information. Participants viewing the baseline drug’s page on Facebook were often able to identify information about the drug’s indications more quickly and easily than participants viewing it on Instagram. That said, participants viewing the pages on Facebook commonly visited the external website to obtain this information. Participants viewing Instagram stimuli frequently attempted (and failed) to decipher the indications based on posted images. For example, one participant viewing Instagram stimuli noted that “Just looking at the page, I’d say weight loss and energy ... They look like they’re on a road trip or rollercoaster, so maybe it’s for the heart or something?” [Mobile, young adult].

In addition to the size of the content, other reasons participants provided for not feeling aware of the drug’s risks and indications after their review included the perception that the page’s information was not sufficiently comprehensive or credible (ie, that it did not mention all relevant risks). Some participants indicated that they would visit the drug’s website for details versus relying solely on the information on any social media page. Several participants (a few on each platform) found scrolling to be a barrier to their efforts to read risk information for understanding. One participant viewing Instagram stimuli explained: “I guess you can also click on the safety story, but I would rather click the link because personally I don’t like pausing to stop the scrolling post. I’m sure it’s slow enough to read, but just a personal preference” [Mobile, young adult]. All participants viewing Facebook pages who voiced frustration with scrolling risk information were older adults.

## Discussion

This unique mixed methods usability study addresses a gap in the literature by enhancing our understanding of consumers’ interactions with and perceptions of prescription drug information on social media pages and posts created by pharmaceutical companies, which is a topic of growing importance, given recent, substantial increases in the use of social media for drug promotion.

### Key Findings and Implications

Observational data revealed that the majority of participants viewed risk information in at least 1 possible location on average across all pages tested during the observation period. However, there was not a single location across the Facebook pages that participants commonly clicked on to view risk information, likely because of high variability in where risk information was located and how it was labeled. Further, we found that the characteristics of the pages themselves can impede efforts to locate and comprehensively review information. With third-party multilink services or when an external link led participants to the full prescribing information, participants did not find the risk information or medication guide. Furthermore, if 2 or more links to an external website were presented together, participants often only clicked the first link even if they subsequently could not find risk information on that website. More consistency across pages and posts may help consumers find drug information.

Although thematic analysis showed that most participants were able to identify the official pages and risk information for each drug, auto-scrolling text and text size posed barriers to identification and comprehensive review for some participants. Participants sometimes reported that risk information was scrolling at a suboptimal speed or presented in a font size that was too small to be read. Occasionally, part of the text was obscured due to inadequate margins. However, a video with scrolling risk information attracted more views than other features. We found that risk information on Facebook pages was consistently viewed more frequently on a laptop than on a mobile device, suggesting that having more screen real estate may make reviewing risk information easier. On Instagram, at least half of the participants consistently clicked on the highlighted story with risk information across the pages. These findings indicate that the layout and formatting of pages and posts can also affect participants’ access to drug information.

Prior research has shown that when consumers do successfully find and read the information on pharmaceutical companies’ social media pages, an assortment of other issues can come into play that may impede the use of that information, such as a lack of quality or reliability, information overload, and the inability to correctly apply information found on the web to the users’ personal health situation [16]. Along these lines, some participants in our study voiced concerns about the clarity, level of detail, and credibility of the safety information they reviewed during our interviews.

Often social media platforms use a symbol, such as a blue checkmark, to indicate that the account is verified or the page is “official.” We found that older adults were less aware of the meaning of the blue checkmark compared to younger participants. This could mean that older adults struggle to locate official drug pages, making them potentially at greater risk of consuming incorrect information from unofficial sources. We also found that older adults generally had a harder time reviewing auto-scrolling risk information and were also less likely than younger participants to know how to pause the scrolling. Part of the reason older adults struggled may be that they are less savvy at navigating social media because they use this information source less than their younger counterparts [16-18]. In summary, our study demonstrates that meaningful barriers remain to consumers’ effective use of social media as a source of prescription drug risk information. To maximize the potential of social media to effectively convey information about prescription drugs, it is essential that the information be made as easy as possible to find, read, understand, and use.

### Strengths and Limitations

This study had several strengths. First, the study’s mixed methods nature enabled us to achieve a comprehensive understanding of users’ perspectives by synthesizing quantitative observational data with qualitative data from semistructured discussions, follow-ups, and probes while maximizing reliability and validity. Second, this study’s sample was on the larger side for an interview-based usability study, and it included a wide range of age groupings to account for generational differences in digital interactions. Given that the majority of US adults now report using or having used some form of social media [19], a truly representative population for this research should encompass all ages. Finally, we implemented multiple measures to ensure that the stimuli had relevance to the study population and that the interactions were as realistic as possible so that we could assess important differences users may experience, as they navigate this sort of content across different platforms or with different devices. These measures included assigning participants to use the platform and device they typically use for social media, using actual pages and posts current and live at the time of the interview as stimuli, and including stimuli pertinent to the study’s target health condition of diabetes.

This study also had some important limitations. First, the set interview length meant that participants were not able to explore on their own for long periods of time. This likely reduced the opportunity for additional possible insights, especially those concerning discoverability. Second, although our sample included adults of all ages, more research is needed with audiences that vary in terms of other sociodemographic characteristics and levels of health literacy. Third, our use of real-life stimuli means that participants could have had prestudy exposure to promotional content for the selected drugs, either on social media or through some other format, such as television advertisements. Finally, it remains unclear how the findings may be applied to other drugs, medical conditions, and social media platforms beyond the small number included in this study.

### Future Research

The exploratory nature of the study provides valuable insights into avenues for further research into the UX of those interacting with prescription drug information on social media. Further exploration with participants who have (or care for those with) other health conditions is likely to provide even more insight into consumer engagement with prescription drug promotion on social media. Since this research tested stimuli only on Facebook and Instagram, supplementary research investigating other widely used platforms could also prove valuable for compiling a well-rounded view of UX with prescription drug promotion in social media.

Additionally, the findings of this study could provide a foundation for developing hypotheses testable by other study types. For instance, experimental stimuli could be developed and tailored to manipulate the way in which the same risk information is presented to enable the quantitative assessment of the level of influence on risk recall and comprehension. Quantitative data such as these could facilitate the development of best practices around UI or UX design features, which could in turn help consumers identify and review important information about drugs on social media more easily. This type of study would circumvent some complexities associated with conducting research in the context of the ever-changing and individualized nature of social media.

### Conclusions

This study contributes to existing research by examining how consumers interact with, interpret, and react to prescription drug promotion on social media. Our findings identified UI or UX design features (including where and how drug risk information is presented) that facilitate or pose barriers to users’ identification, review, and comprehension of the risk information provided on or via prescription drugs’ Facebook and Instagram pages and posts. Based on this study’s findings, we have identified future research possibilities to further explore consumer experience regarding prescription drug promotion on social media, as the information shared on these platforms could have a large impact on consumers’ treatment decisions.

## Abbreviations

UI: user interface
UX: user experience

## Data Availability

The datasets generated and analyzed during this study are available from the corresponding author on reasonable request.
